# Mechanical and Microstructural Characterization of Quarry Rock Dust Incorporated Steel Fiber Reinforced Geopolymer Concrete and Residual Properties after Exposure to Elevated Temperatures

**DOI:** 10.3390/ma14226890

**Published:** 2021-11-15

**Authors:** Muhammad Ibraheem, Faheem Butt, Rana Muhammad Waqas, Khadim Hussain, Rana Faisal Tufail, Naveed Ahmad, Ksenia Usanova, Muhammad Ali Musarat

**Affiliations:** 1 Department of Civil Engineering, University of Engineering and Technology, Taxila 47050, Pakistan; Ibrahim.123456@yahoo.com (M.I.); rana.waqas@uettaxila.edu.pk (R.M.W.); hussainkhadim173@gmail.com (K.H.); naveed.ahmad@uettaxila.edu.pk (N.A.); 2 Department of Civil Engineering, Wah Campus, COMSATS University Islamabad, Wah Cantt 47040, Pakistan; faisal.tufail@ciitwah.edu.pk; 3 Department of Construction of Unique Buildings and Constructions, Peter the Great St. Petersburg Polytechnic University, 195291 St. Petersburg, Russia; plml@mail.ru; 4 Department of Civil and Environmental Engineering, Universiti Teknologi PETRONAS, Bandar Seri Iskandar 32610, Malaysia

**Keywords:** microstructural characterization, quarry rock dust, geopolymer concrete, steel fibers, workability, mechanical strength, elevated temperature, residual compressive strength

## Abstract

The purpose of this research is to study the effects of quarry rock dust (QRD) and steel fibers (SF) inclusion on the fresh, mechanical, and microstructural properties of fly ash (FA) and ground granulated blast furnace slag (SG)-based geopolymer concrete (GPC) exposed to elevated temperatures. Such types of ternary mixes were prepared by blending waste materials from different industries, including QRD, SG, and FA, with alkaline activator solutions. The multiphysical models show that the inclusion of steel fibers and binders can enhance the mechanical properties of GPC. In this study, a total of 18 different mix proportions were designed with different proportions of QRD (0%, 5%, 10%, 15%, and 20%) and steel fibers (0.75% and 1.5%). The slag was replaced by different proportions of QRD in fly ash, and SG-based GPC mixes to study the effect of QRD incorporation. The mechanical properties of specimens, i.e., compressive strength, splitting tensile strength, and flexural strength, were determined by testing cubes, cylinders, and prisms, respectively, at different ages (7, 28, and 56 days). The specimens were also heated up to 800 °C to evaluate the resistance of specimens to elevated temperature in terms of residual compressive strength and weight loss. The test results showed that the mechanical strength of GPC mixes (without steel fibers) increased by 6–11%, with an increase in QRD content up to 15% at the age of 28 days. In contrast, more than 15% of QRD contents resulted in decreasing the mechanical strength properties. Incorporating steel fibers in a fraction of 0.75% by volume increased the compressive, tensile, and flexural strength of GPC mixes by 15%, 23%, and 34%, respectively. However, further addition of steel fibers at 1.5% by volume lowered the mechanical strength properties. The optimal mixture of QRD incorporated FA-SG-based GPC (QFS-GPC) was observed with 15% QRD and 0.75% steel fibers contents considering the performance in workability and mechanical properties. The results also showed that under elevated temperatures up to 800 °C, the weight loss of QFS-GPC specimens persistently increased with a consistent decrease in the residual compressive strength for increasing QRD content and temperature. Furthermore, the microstructure characterization of QRD blended GPC mixes were also carried out by performing scanning electron microscopy (SEM), X-ray diffraction (XRD), and energy dispersive spectroscopy (EDS).

## 1. Introduction

There has been a significant increase in construction activities around the globe to fulfill the growing infrastructural needs. Ordinary Portland cement concrete (OPC) is the most important material generally used in all construction activities. The ordinary Portland cement is manufactured by the consumption of fuel and conversion of raw materials, during which an enormous amount of CO_2_ is released into the atmosphere. According to a study, one ton of carbon dioxide is released into the environment during the production of one ton of cement [1]. Further, an enormous amount of waste is produced from different industries, such as slag from steel or iron industries, ceramic wastes from ceramic industries, red mud from alumina industries, and fly ash from thermal power plants. It is challenging for researchers and environmentalists to find an alternative to traditional OPC concrete and manage or dispose of these industrial wastes. Therefore, it is necessary to find the best solutions to effectively utilize these industrial byproducts/wastes to minimize land and air pollution. One of the solutions is alkali-activated cement or geopolymer concrete (GPC) that is produced by alkali activation of different byproducts/waste materials and minerals, i.e., fly ash, slag, rice husk ash (RHA), waste ceramic materials, etc. [2,3]. A review of alternative binders reveals that gypsum, geopolymer, and starch can be good alternatives to lime and magnesium-based binders for building materials made of bio-composites [4]

GPC is one of the better and feasible solutions to decrease or completely avoid using traditional ordinary Portland cement concrete. Further, it promotes industrial wastes/byproducts to produce environmentally friendly binder material [2,3]. Due to the early compressive strength, better chemical resistance, and low permeability, GPC has presented itself as a good alternative to the traditional binders [5,6]. It can be manufactured from basic geological materials (such as metakaolin) or industrial pozzolanic materials (slag, fly ash, RHA, and ceramic wastes), which consist of a large amount of alumina (Al_2_O_3_) and silica (SiO_2_) [7,8,9,10]. Since SiO_2_ and Al_2_O_3_ are the main oxides in the GPC production, industrial waste materials such as fly ash, slag, copper, and zinc SG can be used as an aluminosilicate source in GPC synthesis. The FA has been widely used to produce geopolymer binders due to its wide availability, durability, and high pozzolanic properties [11,12,13,14,15]. Several studies highlight low calcium FA-based GPC production under elevated temperature curing for short periods [16,17,18]. However, the results are not promising under ambient curing conditions due to the slower polymerization process. The polymerization process leads to the formation of calcium aluminate silicate hydrate and sodium aluminate silicate hydrate compounds [19]. It was found that the optimum curing temperature was 60 °C for a curing duration of 19 to 24 h, depending on the type of binder contents for the activation of the polymerization process in GPC. Different structural members indicate better mechanical properties of GPC with inclusion of steel fibers and binders [20]. It was also concluded in the previous studies that heat curing limits the usage of GPC to precast structural members only. Therefore, it is imperative to study the feasibility of using ambient cured cast-in-situ GPC. Researchers have endorsed the use of alccofine in GPC to achieve encouraging results at ambient curing conditions [19]. Some researchers have also tried to enhance the reactivity of FA in the basic environment (i.e., at ambient temperature) by increasing the fineness of FA [21] and by the addition of calcium-containing materials [22,23] such as SG [24], alccofine [19], etc. It has been reported that SG blended FA-based GPC specimens showed good mechanical properties, and resistance to elevated temperature [25,26] and sodium sulphate attack. However, it showed substantial deterioration in magnesium sulphate attack [27] and exhibited increased shrinkage [28]. Several studies have been carried out to investigate the effect of SG on the fresh, mechanical, and durability properties of GPC. However, the development of GPC mixes by blending quarry rock dust (QRD) wastes as a binder has rarely been explored. The QRD is a waste material of rock quarries that is produced during the coarse aggregates manufacturing process [29]. A portion of this unwanted waste is often used on site as a filling material for the quarry pit [30]. Recently, QRD has been used in GPC and OPC specimens as a partial replacement of fine aggregates, i.e., sand [29,31]. QRD has also been used as a partial replacement of cement in conventional concrete and showed improved strength results with 20% replacement of QRD with the OPC. The effect of QRD as a binder on properties of FA and SG-based GPC at ambient as well as at elevated temperatures has also been recently investigated [32] and used in columns with steel fibers. The columns were tested under concentric and eccentric loading [33]. The compressive strength of control OPC specimens (without QRD) was 50.23 MPa; whereas the compressive strength of OPC mixtures with 10%, 20%, and 30% replacement of QRD were increased by 4.8%, 8.2%, and 5.92%, respectively. Similarly, the flexural strength of control specimen was 5.12 MPa and it was increased by 107%, 110%, and 106% for 10%, 20%, and 30% replacement of QRD, respectively, at 28 days [30]. It therefore would be interesting to explore the suitability of QRD as a binder in GPC mixes which is the objective of the present study.

GPC is a promising material for the construction industry with environmental benefits and equally good engineering properties. However, one of the drawbacks of GPC in large-scale structural applications is the low ductility [34,35]. There are different types of fibers, i.e., steel, nylon, polypropylene, and polyethylene, that can improve the ductility, flexural, and tensile properties of GPC blends. However, steel fibers (SF) have been the best due to their better ductile and thermal properties at elevated temperatures [36,37]. Genesa et al. [35] explored the basic characteristics of SF-based GPC. It was observed that compressive as well as splitting tensile strength was increased by a margin of 8.5% and 61.6%, respectively, with the fiber volume fraction of 1%. It has also been reported that the splitting tensile and flexural strength of GPC mixes with 0.5% steel fibers by volume were increased by 19–38% and 13–44%, respectively, than the plain samples [38]. Another study reported that compressive and flexural strengths were increased by 3.4% and 31.5%, respectively, by adding 1.2% by volume SF [39].

Recently, research proved that GPC has potential to be used as thermal barriers [40,41]. The structure may be exposed to open fire as well as closed fire, so it must possess thermal stability and fire resistance. The literature shows that fiber-reinforced geopolymers can be excellent materials for thermal and fire-resistant applications [40,42].

The actual behavior of concrete exposed to elevated temperatures depends on many factors such as the properties of materials, heating rate, maximum temperature, exposure period, cooling method, and loading level at the time of cooling [43,44]. It has been found that SF has shown a higher retaining capacity of its original mechanical properties during fire due to its higher melting temperature [45]. It has also been reported that GPC specimens have good resistance against fire due to the presence of nanopores in abundance that allows bonded water to migrate and evaporate without destroying the aluminosilicate network [5,46,47]. Investigations proved that GPC loses its strength after being exposed to a temperature of about 400 °C depending on the raw material. The drop in compressive strength (in percentage) of fly-ash-based GPC is 35%, 44%, 50%, and 75% when exposed to elevated temperatures, i.e., 400 °C, 600 °C, 800 °C and 1000 °C, respectively [48]. Similar to fire resistance, GPC specimens also have frost resistance [49].

Currently, many countries are facing land and air pollution problems. A huge amount of industrial wastes/byproducts are produced globally. The disposal of these wastes in dump yards is linked with high costs and a negative impact on the environment. There is a need to work on creating better and feasible solutions that can productively use industrial wastes. The QRD wastes, also known as limestone dust, dolomite or silica powders, are produced during the quarrying of the large parent mass rock to produce aggregates. These wastes are nonbiodegradable and cause environmental pollution creating health hazards. Therefore, it is best if such wastes can be recycled and used in construction activities in order to help preserve natural resources and the environment.

Several studies are available on fiber-reinforced FA and SG-based GPC at ambient and heat curing conditions [18,24,50]. However, the publications on QRD as a geopolymer binder in fly ash and slag-based GPC are rather scarce. Therefore, the present study has been undertaken to examine the properties of fiber-reinforced fly ash and slag based GPC mixtures with QRD incorporation at room and elevated temperatures. The effects of QRD incorporation on fresh, mechanical, and microstructural properties have been investigated. The optimum percentage of QRD addition has been worked out considering the performance in workability and mechanical properties. Moreover, weight loss and residual compressive strength were also investigated after heating the specimens at elevated temperatures, i.e., 400 °C and 800 °C.

## 2. Materials and Methods

### 2.1. Experimental Program

**Figure 1:**
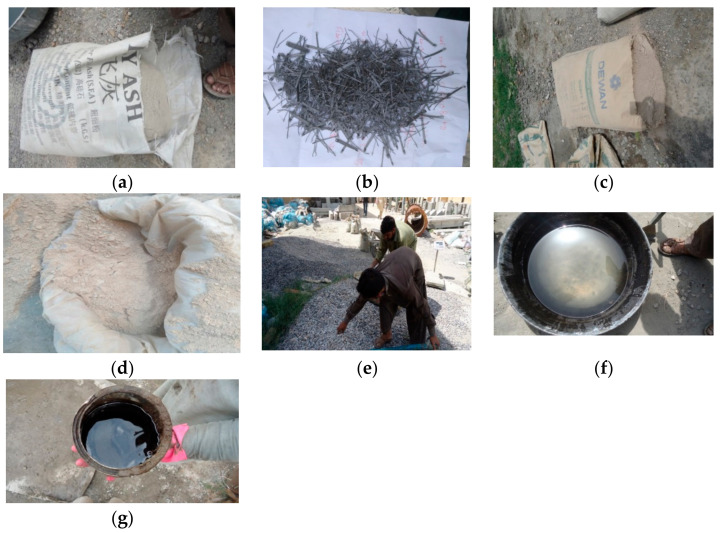
The materials used in the present study to produce QRD incorporated FA and SG-based GPC (QFS-GPC) reinforced with steel fibers; (**a**) fly ash, (**b**) ground granulated blast furnace slag, (**c**) steel fibers, (**d**) quarry rock dust at site, (**e**) aggregates, (**f**) alkaline solution, and (**g**) superplasticizer.

An experimental program was designed to achieve the objective of finding an optimum mix of ambient cured, ternary blended GPC comprising fly ash, slag and QRD, reinforced with steel fibers. The low calcium FA (Super fine ash, Matrixx, Karachi, Pakistan), SG (ground granulated blast furnace slag Grade-80, Dewan Cement (PVT) Limited, Karachi, Pakistan), and QRD were utilized as a binder to produce GPC mixtures. The QRD wastes were obtained from the Margallah hills quarries near Taxila, Pakistan. The QRD is collected at the bottom of aggregate crushers during the formation of the coarse aggregates. It was grinded to achieve the required size equivalent to the OPC particles, which can be sieved through a 45 µm sieve. The OPC type II cement conforming to ASTM C-150 [51] was used for control specimens of conventional concrete, the properties of which are provided in Table 1. The fly ash (FA) was collected from Karachi, Pakistan through combustion process of coal (steam coal) in thermal power plants. Table 2 shows the chemical composition of FA, SG, and QRD, determined from X-ray fluorescence (XRF) analysis (Fecto cement factory, Taxila, Pakistan). The alkaline activator used in this study consists of sodium silicate and sodium hydroxide. The molarity of sodium hydroxide was kept as 12M. It was prepared one day before the application by mixing 98% pure flakes with tap water. The sodium silicate solution has a modulus ratio (MR) of SiO_2_ to Na_2_O between 1.90 and 2.01. The chemical composition of sodium silicate is shown in Table 3. The local natural river sand was used as fine aggregates. Crushed stone aggregates available locally in the size of 10 mm and 20 mm was used as a coarse aggregate. The fineness modulus of coarse aggregate conformed to ASTM-C136-06 and specific gravity satisfied ASTM-C127-07. The coarse aggregates (CA) were obtained from the Margallah hills quarry near Taxila, Pakistan. Table 4 shows the properties of coarse and fine aggregates. The commercially available hooked end hard-drawn wire SF (MasterFiber^®^ S 65, BASF, Karachi, Pakistan), conforming to ASTM A820 [52], type 1, were used to improve tensile as well as flexural strength of the GPC. The SF provided best results under impact load than the remaining fibers. The specifications of hooked end steel fibers are presented in Table 5. The alkaline solution used in GPC mixes has a sticky characteristic. Therefore, their use makes the GPC mixes more viscous than the ordinary concrete. A naphthalene-based superplasticizer (SP) (Chemrite-SP 200, Imporient Chemicals (PVT) LTD, Lahore, Pakistan) confirming to ASTM C494 was used to increase the workability of GPC mixes [53]. The different materials used in this study are shown in Figure 1.

Table 6 shows a total of 18 mixtures were designed with different proportions of QRD (0%, 5%, 10%, 15%, and 20% by weight of binder) and steel fibers (0.75% and 1.5% by volume). The SG was replaced by different proportions of QRD in FA, and SG-based GPC mixes to study the effect of QRD incorporation. As shown in Table 7, three mix types comprising OPC concrete group serving as the control mix group; another group GPC-A of three GPC mixes without QRD; while the remaining four GPC groups viz. GPC-B, GPC-C, GPC-D, and GPC-E, with 5%, 10%, 15%, and 20% QRD, partially replacing SG (by weight of binder) and keeping all the other ingredients the same in the groups. Further, each group comprises three mix types with 0%, 0.75%, and 1.5% (by volume of composites) SF, thus making a total of 18 mix types in the six groups.

### 2.2. Mixing and Casting Procedure

The mixtures were prepared based on a unit volume of one cubic meter. The quantity of binder content was kept fixed at 400 kg/m^3^ in all the mixes. A total of eighteen mixtures were designed: three OPC-based concrete as control specimens and fifteen GPC specimens with fly ash, slag, and QRD as the source binding materials. The amount of FA was kept constant at 200 kg/m^3^ in all the GPC mixes, whereas SG was replaced with QRD at 0%, 5%, 10%, 15%, and 20% by weight of the binder. A total of 540 specimens were cast, consisting of 270 cubes (150 × 150 × 150 mm) for compressive strength, weight loss, and residual compressive strength tests; 162 cylinders (150 mm dia, 300 mm height) for splitting tensile strength tests; and 108 prisms (100 × 100 × 500 mm) for flexural strength tests. The alkaline activator solution was kept at 200 kg/m^3^ with a sodium silicate to sodium hydroxide ratio of 1.5 and alkaline solution to binder ratio (A/B) of 0.5 in all GPC mixes. The steel fibers with both ends hooked were used in the mixes with varying contents of 0.75% and 1.5% by volume of the concrete. The specifications of mix design proportions and mix designations are shown in Table 6 and Table 7.

**Figure 2:**
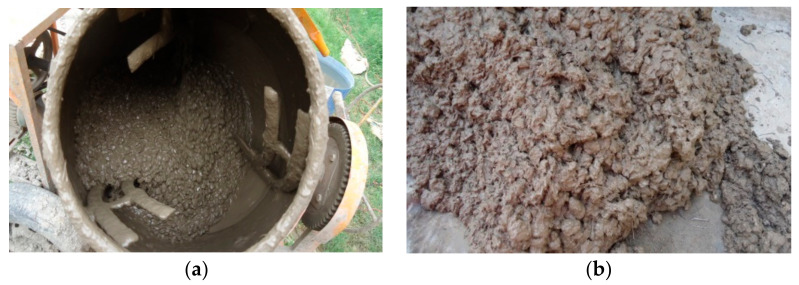
(**a**) The mixing of ingredients in a mechanical mixer. (**b**) The freshly prepared SF-reinforced GPC mixture.

**Figure 3:**
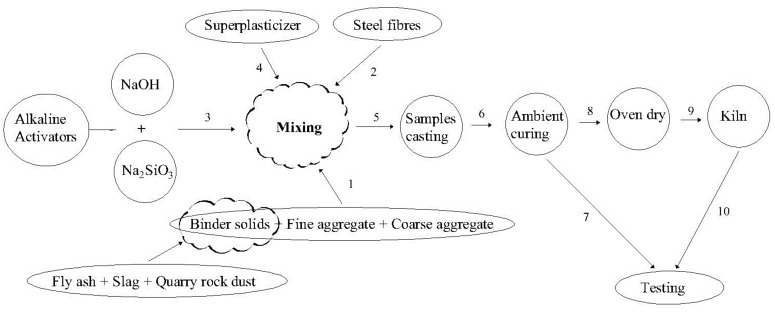
The mix production to the testing sequence of GPC specimens. 1—mixing of Raw materials; 2—adding Steel fibers; 3—Adding alkaline solution; 4—Adding superplasticizer; 5—Casting of samples; 6—Curing of samples at room temperature; 7—Compressive, tensile and flexural testing of samples; 8—Oven dry of samples; 9—Heating of samples at elevated temperature; 10—Mechanical tests.

All the mixes were prepared in a mechanical mixer of 0.15 m^3^ capacity, as shown in Figure 2a. Before mixing ingredients, aggregates were prepared to the saturated surface dry (SSD) condition. The sodium hydroxide solution was blended a day before [19] the application and mixed with SS solution about 30 min before its use [24] to improve the reactivity of the solution. Firstly, the coarse and fine aggregates and binders (fly ash, slag, and QRD) were mixed in dry condition thoroughly in the mixer for 2 min. The SF was then added to the dry mixture, and mixing was continued for another 2 min, ensuring adequate and homogenous dispersion of fibers in the mix. After that, the premixed alkaline solution was incorporated gradually into the mixer. Then, mixing was continued for another 2–3 min to achieve a uniform homogeneous mixture. Finally, superplasticizer and remaining water were added in the mix to achieve the required workability in the range of 50–89 mm and mixing was continued for another 2–3 min. The freshly prepared steel fibers reinforced GPC mix is shown in Figure 2b. The flow chart elaborating the mixing sequence of GPC mixes is shown in Figure 3.

The newly mixed concrete was instantly cast into different molds, i.e., cylinders, cubes, and prisms. All specimens were placed in a room at ambient temperature. After 24 h, all the samples were demolded and kept in the ambient curing conditions for 7, 28, and 56 days. Three specimens were used for testing each mix, and an average result was reported. The designated specimens (150 mm cubes) were exposed to elevated temperatures (400 °C and 800 °C) after 56 days of curing to investigate the weight loss and residual compressive strength. Before placing the samples in the kiln, they were dehydrated in an oven for 24 h at 105 ± 5 °C to remove any free water, thus preventing the samples from a possible explosion in the kiln during the heating procedure due to very high pore water pressure resulting from the superheated water.

### 2.3. Experimental Setup

A series of tests were carried out to determine the fresh properties (workability), mechanical properties (compressive, split tensile, and flexural strengths), residual properties after exposure to elevated temperature (weight loss and residual compressive strength), and microstructural properties (from X-ray diffraction analysis, scanning electron microscope images, and energy dispersive spectroscopy). Workability is defined as the ease of placement and compaction of freshly mixed concrete. The slump cone test is commonly used to check the workability of freshly made concrete conforming to ASTM C143M-15a [54] The mechanical properties of specimens, i.e., compressive, splitting tensile, and flexural strengths were determined by testing cubes, cylinders, and prisms, respectively, at different ages (7, 28, and 56 days). The specimens were heated to 800 °C to evaluate the resistance of specimens to elevated temperature in terms of residual compressive strength and weight loss. The microstructural properties from scanning electron microscopy (SEM), X-ray diffraction (XRD), and energy dispersive spectroscopy (EDS) were investigated.

A universal testing machine (UTM) of 3000 KN capacity was used for compressive and splitting tensile strength tests of the specimens at various ages, i.e., 7, 28, and 56 days, according to ASTM C39/C39M-03 [55] and C496/C496M−11, respectively. The flexural strength test was performed on prismatic samples at the ages of 28 and 56 days under third point loading according to ASTM C1609/C1609M-19a [56]. The workability of mixes was measured by using a slump cone test conforming to ASTM C143M-15a [54]. After 56 days of curing, three samples (150 mm cubes) from each mix group were subjected to elevated temperatures (400 °C and 800 °C) in an automatic controlled electric furnace of 1000 °C capacity following ASTM E 119 [57]. The samples were then cooled at room temperature, and residual properties, i.e., compressive strength and weight loss, were determined. The microstructural characterization of the specimens was evaluated by X-ray diffraction (XRD) analysis, energy dispersive spectroscopy (EDS), and scanning electron microscopy (SEM). The XRD test was performed to provide fundamental information on the geopolymer crystal structure. A crystal structure is one of the important aspects of materials since many properties depend on it. The X-ray diffraction (XRD) test was performed using JEOL JDX-3532 (JEOL, Tokyo, Japan), with a step size of 0.025° and 2θ range from 10–75° to analyze the geopolymer structure to find whether it is crystalline or amorphous. The powder method was used to evaluate the degree of crystalline structure in the polymer. The sample was scanned with a voltage of 40 kV and a current of 30 mA using a copper X-ray tube (Cu-Kα radiation). It has been reported that diffraction occurs only if electromagnetic radiation interacts with periodic structures. The non-crystalline portion in GPC mixes simply scatters the X-ray beam to give a continuous background, whereas the crystalline portion causes diffraction lines (peaks) that are not continuous. Miller indices of the peaks describe the information about the planes of diffraction [19,58].

The EDS test was carried out to study the chemical composition of the GPC mixtures. The energy dispersive spectroscopy (EDS) test was performed for the area using JSM-5910 (Oxford Instruments, Abingdon, UK) to find the chemical composition of geopolymers. For the purpose of testing, a sample of GPC is taken out from the middle of the cube specimen cured for 28 days and then ground to a powder with the help of mortar and pestle. The powdered sample is then oven-dried to remove moisture. The EDS data is acquired using INCA software at five different spectrums (locations) on the SEM image. SEM was undertaken to study the fracture surface of the GPC specimens. The SEM analysis was carried out using JSM-6490 (JEOL, Tokyo, Japan) to study the microstructural behavior of GPC specimens when subjected to an elevated temperature of 800 °C. Samples were collected from the failed specimens in the compressive strength test from the adjacent parts of failure surfaces.

## 3. Results and Discussion

### 3.1. Workability

**Figure 4:**
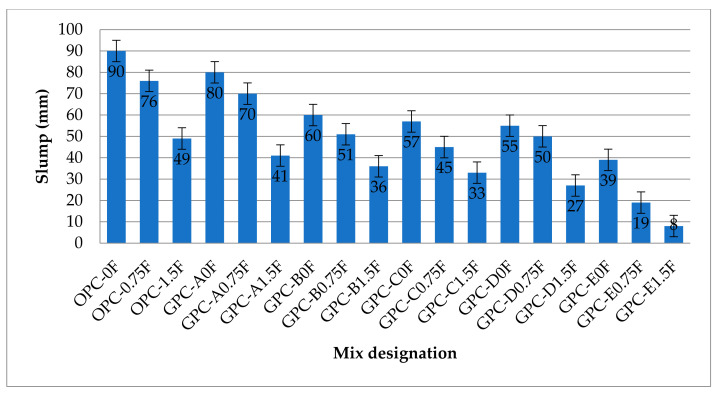
The slump values of GPC and OPC mixes.

The slump values of OPC- and QRD-blended FA-SG-based GPC (QFS-GPC) mixtures are shown in Figure 4. It can be observed from Figure 4 that all GPC mixtures without steel fibers (GPC-A0F, GPC-B0F, GPC-C0F, GPC-D0F, and GPC-E0F) have lower slump values than OPC-based mixes due to the combined effect of slag and QRD particles along with higher viscosity of alkaline solutions. It can also be observed that QRD content has a negative effect on the workability of GPC mixes. The workability of QFS-GPC specimens persistently decreased with the increase in QRD content from 0% to 20%. The slump values of QFS-GPC mixes, i.e., GPC-B0F, GPC-C0F, GPC-D0F, and GPC-E0F are 25%, 29%, 31%, and 51% lower, respectively, than their counterpart without QRD, i.e., GPC-A0F. Previous studies have also reported this decreasing trend of the slump with the increased QRD [30]. This decreasing trend of slump can be attributed to the angular shape particles of QRD [59] that restrain the flowability of the mixture, contrary to the spherical-shaped particles of FA [34] that make concrete more flowable. The workability of GPC mixes was observed to be lesser than the corresponding OPC-based mixes. The slump values of GPC mixes viz. GPC-A0F, GPC-B0F, GPC-C0F, GPC-D0F, and GPC-E0F were 11%, 33%, 37%, 39%, and 57% lower than the OPC-based control mix. It has been investigated that workability of FA-based GPC decreases by increasing SG content [24].

It can be noticed from Figure 4 that the workability of steel-fibers-reinforced concrete mixtures is lower than their counterparts, i.e., plain specimens (without fibers). The slump values of OPC-0.75F and OPC-1.5F are 16% and 36% lower, respectively, than OPC-0F (without steel fibers). Similarly, the slump values of GPC mixes, GPC-A0.75F and GPC-A1.5F, are 13% and 41% lower than their counterpart plain samples of GPC-A0F. The trend of decreasing workability in GPC mixes increases with the increase in QRD content. The slump values of group E mixes, i.e., GPC-E0.75F and GPC-E1.5F, are 51% and 58% lower than GPC-E0F. The addition of SF with 1.5% by volume decreased the workability up to 60% then 0.75% by volume of SF. This can be due to uneven scattering of fibers that may have hindered the movement of mixture particles. Moreover, the fibers absorb more binder (cement, FA, SG, or QRD) mortar due to the large surface area, which increases the viscosity of mixes resulting in low slump values. Therefore, the optimum SF content from the above finding is 0.75% considering workability.

The rheology of a GPC mix is generally not similar to that of an OPC mix. Hence, the slump values of GPC do not resemble the same level of workability in OPC mixtures [24]. Based on compaction, slump values of GPC are classified as: highly workable (90 mm and above), medium workable (50–89 mm), and low workable (less than 50 mm) [56]. According to this criterion, GPC mixtures with 0% and 0.75% SF contents are medium workable except GPC-E0F; and those with 1.5% SF content are classified as low workable.

### 3.2. Compressive Strength

**Figure 5:**
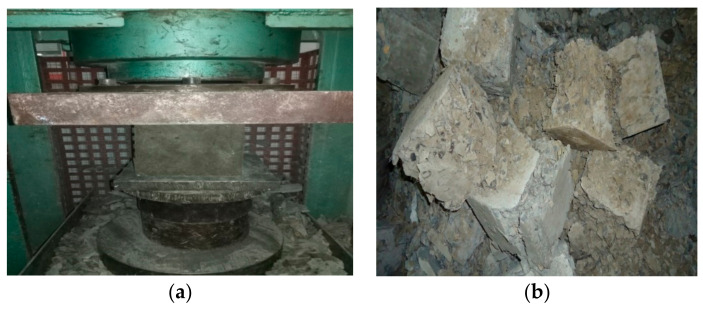
(**a**) The compressive testing of cubes; and (**b**) the failure samples.

**Figure 6:**
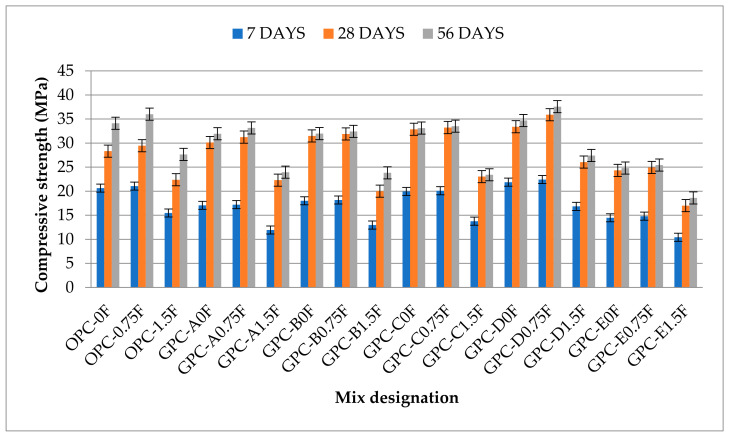
The compressive strength values of GPC and OPC mixes.

Compressive strength is an important property of concrete that is connected to other mechanical properties as well. According to ACI 318 M-11 [59], the 28 day compressive strength needs to be at least 28 MPa for basic engineering applications, while it should be 35 MPa for corrosion protection of deform steel bars in concrete. In this study, all samples were tested at the age of 7, 28, and 56 days according to ASTM C39/C39M [55], as shown in Figure 5. The mean values of the compressive strength test results of OPC and GPC mixes obtained from three identical samples are shown in Figure 6. Generally, FA- and SG-based GPC mixes exhibit higher compressive strength values than the OPC-based mixes [16]. It can be observed from Figure 6 that the compressive strength of GPC mixes GPC-B0F, GPC-C0F, and GPC-D0F, increased by increasing the QRD replacement level up to 15% (i.e., for 5%, 10%, and 15% QRD replacement).

The 28 day compressive strength of QFS-GPC mixes viz. GPC-B0F (5% QRD), GPC-C0F (10% QRD), and GPC-D0F (15% QRD) are 5%, 9%, and 11% higher than the GPC-AOF (without QRD). This increase in strength due to QRD addition can be attributed to the increased quantity of lime produced in the geopolymerization process due to SG replacement with QRD. From the XRF analysis of SG and QRD, CaO content in SG is 37.33%, whereas QRD composes 47.13% of CaO content as shown in Table 2. The replacement of SG with QRD ultimately resulted in increasing the CaO contents in the geopolymer matrix. It has been reported that calcium-containing materials such as SG, alccofine, and QRD accelerate the rate of polymerization at ambient temperature (room temperature) and reduce the pore sizes [60]. The inclusion of the calcium-containing materials has increased the compressive strength of the QRD blended FA-SG-based GPC (QFS-GPC) mixes. Hence, GPC mixes with QRD replacement level up to 15% would produce compacted geopolymer matrix compound, which will increase the compressive strength of the specimens at early ages. However, when the amount of QRD is increased further from 15% to 20% as in mix GPC-E0F, it decreases the workability of the mix drastically as shown in Figure 4, making it difficult to handle during placement. Hence, extra water or superplasticizer was added to the mix GPC-E0F to increase the workability that ultimately resulted in decreasing the compressive strength by an amount of 19% compared with GPC-A0F (without QRD content). This phenomenon has also been reported by Hake et al., 2018 [61].

The 28 day compressive strength of QFS-GPC mixes viz. GPC-B0F, GPC-C0F, and GPC-D0F are 11%, 16%, and 18% higher than the control OPC mix (OPC-0F). However, the compressive strength of GPC-E0F is 14% lower than the OPC-0F. After 28 days, the compressive strength of GPC-D0F (33.4 MPa) is almost 18% higher than the control mix OPC-0F. Therefore, mix GPC-D0F can be considered as an optimum mixture without any fiber reinforcement considering the compressive strength.

It was observed that the effect of SF addition on the compressive strength is very low compared with the flexural and tensile strengths. The compressive strengths of GPC and OPC mixes were increased in the range of 2–8% by adding 0.75% steel fibers (by volume) than their counterparts (without steel fibers). When the fraction of SF was further increased from 0.75% to 1.5% in all GPC and OPC mixtures, the strength was further decreased by 20–30% of the counterparts without fibers. This decrease in strength can be due to the uneven dispersion of fibers causing insufficient compaction and non-uniformity of the mix.

### 3.3. Splitting Tensile Strength

**Figure 7:**
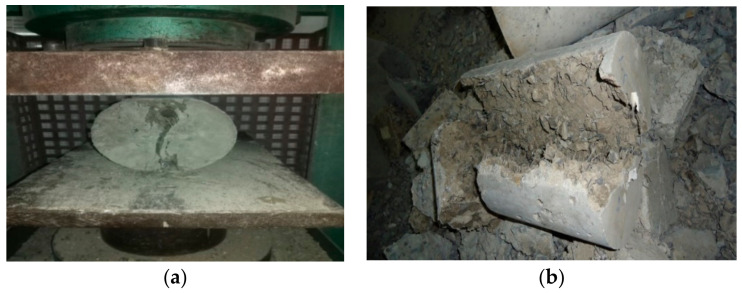
(**a**) The splitting tensile load application on a cylindrical specimen; and (**b**) the failure of the specimen after the test.

**Figure 8:**
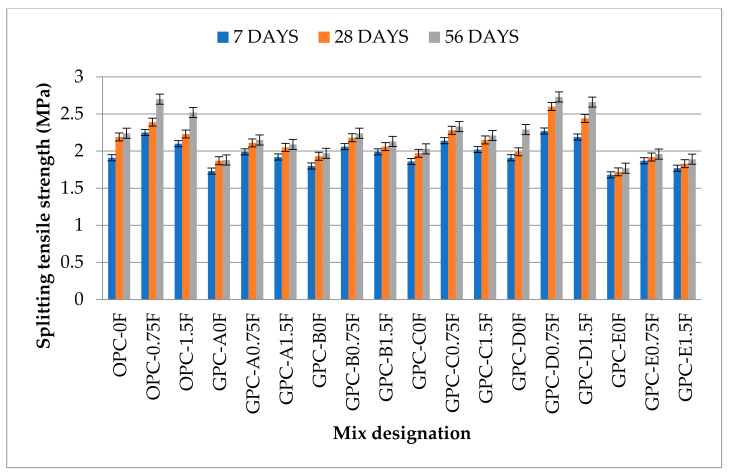
The splitting tensile strength values of GPC and OPC mixes.

It is an important mechanical characteristic of concrete that is used in designing some reinforced concrete structural members. The splitting strength testing setup of cylindrical samples is shown in Figure 7a and determined at the ages of 7, 28, and 56 days according to ASTM C496 [62]. The results of splitting tensile strength values of OPC and GPC specimens are shown in Figure 8.

The splitting tensile strength of OPC-0F (1.91 MPa) and GPC-D0F (1.94 MPa) at the age of 7 days were maximum among OPC and GPC specimens, respectively. The splitting tensile strength of QFS-GPC specimens without fibers viz. GPC-B0F, GPC-C0F, and GPC-D0F are 3%, 5%, and 6% higher, respectively, than the GPC control mix GPC-A0F (without QRD). However, GPC-E0F shows a decrease in splitting tensile strength than GPC-A0F. This decrease in strength can be attributed to the decreased workability of the mix due to increased QRD content, thus making it more difficult to handle during placement. The additional water or admixture (superplasticizer) was added to the mix (GPC-E0F) to increase the workability, ultimately decreasing the splitting tensile strength.

The values of splitting tensile strength of all GPC mixes without fibers, i.e., GPC-A0F, GPC-B0F, GPC-C0F, GPC-D0F, and GPC-E0F, are 15%, 12%, 10%, 9%, and 21%, respectively lower than the control OPC mix OPC-0F; which shows that GPC mixes are weak in tensile strength than the OPC mixes. An increase in splitting tensile strength was also observed with the increase in curing age. The maximum splitting tensile strength in non-fiber mixes was observed for the mix GPC-D0F with values of 2.27 MPa and 2.36 MPa, respectively, at 28 and 56 days. These higher strength values can be due to more compactness in the presence of optimum calcium content than the other non-fiber GPC mixes.

It can be observed from Figure 8 that the addition of SF by 0.75 fraction of volume resulted in increasing the splitting tensile strength of GPC mixes viz. GPC-A0.75F, GPC-B0.75F, GPC-C0.75F, GPC-D0.75F, and GPC-E0.75F by 13%, 13%, 16%, 31%, and 12%, respectively, than their counterparts without fibers. This increase can be attributed to the relatively strong bonding and matrix between the aggregate and SF at 0.75% fraction by volume. The mix GPC-D0.75F achieved the maximum splitting tensile strength with 15% QRD and 0.75% SF. The results are also in agreement with the previous studies [60]. However, the increase in SF content from 0.75% to 1.5% decreased the splitting tensile strength. The possible cause can be uneven dispersion of fibers in the mixes for more than 0.75% SF, which caused low workability of the mixes. Extra water and superplasticizer were used during the mixing procedure resulting in a decrease in the strength.

### 3.4. Flexural Strength

**Figure 9:**
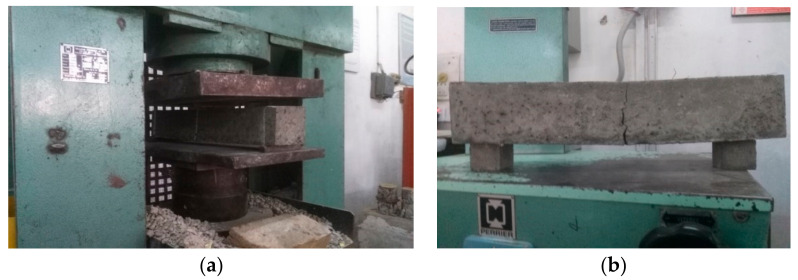
(**a**) Flexural testing setup for Prismatic specimens of length 500 mm; and (**b**) the failure of a specimen after the test.

**Figure 10:**
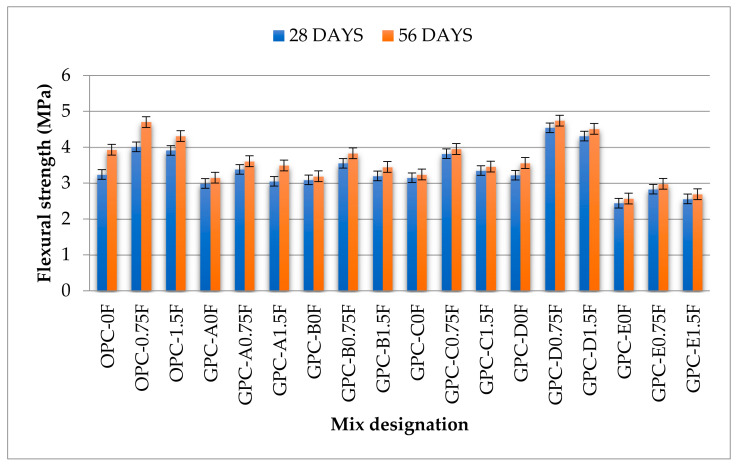
The flexural strength values of GPC and OPC mixes.

The flexural strength test is carried out to find the indirect tensile strength of concrete, also known as the modulus of rupture (MOR). It is an important property that affects concrete’s shear strength, bending characteristics, and brittleness ratio in structural concrete design. In this study, the flexural strength was determined by using prismatic specimens (100 × 100 × 500 mm) at the age of 28 and 56 days according to ASTM C1609 [56], the standard test method used for the flexural performance of fiber-reinforced concrete. The flexural strength testing of prismatic samples under center point loading is shown in Figure 9. It can be noticed from Figure 10 that flexural strength of QFS-GPC mixes viz GPC-B0F, GPC-C0F, and GPC-D0F, are 3%, 7%, and 10% higher, respectively, than GPC-A0F (without QRD). This higher strength indicates that the flexural strength of QRD-blended specimens increased with the increase in QRD content up to 15%. The maximum flexural strength of non-fiber specimens at the age of 28 days was obtained by the mix GPC-D0F (3.65 MPa) with 15% QRD content. However, the flexural strength of GPC-E0F (with 20% QRD content) decreased by 18% from GPC-A0F (without QRD). The test results also showed that flexural strength of GPC mixes without fibers, i.e., GPC-A0F, GPC-B0F, GPC-C0F, and GPC-E0F were 8%, 5%, 2%, and 25% lower than the OPC control mix, i.e., OPC-0F. However, the flexural strength of GPC-D0F was 2% higher than the mix OPC-0F. Hence, GPC-D0F can be considered as an optimum mix considering the flexural strength. The test results also showed that the flexural strength of all GPC mixes increased with age. However, this rate of strength gain is slower than the OPC mix samples.

The addition of SF improved the flexural strength and improved the post-cracking behavior (also called crack bridging effect) for all GPC and OPC mixes. The flexural strength of GPC mixes viz. GPC-A0.75F, GPC-B0.75F, GPC-C0.75F, GPC-D0.75F, and GPC-E0.75F are 13%, 15%, 20%, 38%, and 16% higher than their counterparts without fibers. It was also observed that the increase in fiber content from 0.75% to 1.5% by volume decreased the flexural strength of GPC mixes. This decrease in strength can be due to the uneven dispersion of steel fibers causing insufficient compaction and non-uniformity of the mix. Experimental research proved that the addition of 0.5% SF in oil palm shell (OPS)-based GPC improves flexural strength by approximately 13–44% [38].

### 3.5. Weight Loss and Residual Compressive Strength

**Figure 11:**
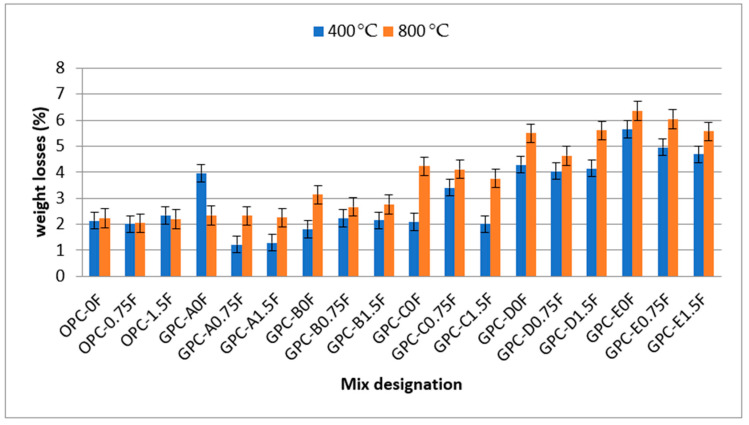
The weight loss values of GPC and OPC mixes after heating the specimens at elevated temperature.

The specimens from each mix group were subjected to elevated temperatures (400 °C and 800 °C) at the age of 56 days to measure the weight loss and residual compressive strength. The oven-dried samples were placed in an electric furnace of 1000 °C heating capacity. The specimens were exposed to an elevated temperature at 8 °C/min heating rate until the target temperature was reached. The specimens were kept for 1 h at the required temperature, i.e., 400 °C and 800 °C. After heating, the specimens were cooled at room temperature. The weight before and after the exposure was measured to determine the weight loss of specimens. The results for weight loss of specimen mixes are presented in Figure 11. The replacement of SG with QRD resulted in an increase in the weight loss of QRD-blended mixes. The weight loss observed in GPC mixes viz. GPC-A0F, GPC-B0F, GPC-C0F, GPC-D0F, and GPC-E0F at 800 °C were 2.34%, 3.13%, 4.23%, 5.5%, and 6.35%, respectively. The weight loss values were increased with the increase in QRD content. The increase in weight loss may be due to the higher loss on ignition (LOI) of QRD (38.65%) compared with the FA (2.9%) and SG (3.4%). The weight loss values of QRD-blended GPC mixes are higher at 800 °C than 400 °C since the release of gases in the form of carbon dioxide (CO_2_), due to thermal decomposition of concrete, occurred at 800 °C. The addition of 0.75% steel fibers in all GPC mixes, i.e., GPC-A0.75F, GPC-B0.75F, GPC-C0.75F, GPC-D0.75F, and GPC-E0.75F, resulted in a reduction in weight loss by an amount of 2.32%, 2.67%, 4.11%, 4.63%, and 6.04%, respectively, compared with their counterparts without fibers. This reduction in weight loss by fibers can be due to the high heat-absorbing capacity of SF. The lowest weight loss was observed in the OPC mix OPC-0.75F and the GPC mix GPC-A0.75F. The reduced weight loss in these specimens can be due to lesser calcium content and SF presence that resisted the decomposition process.

**Figure 12:**
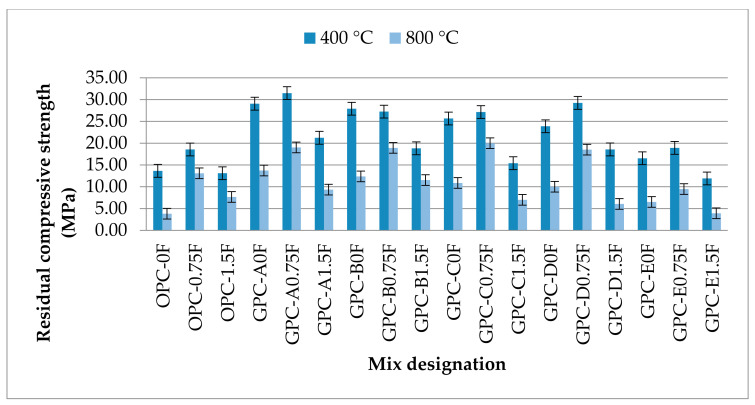
The residual compressive strength values of GPC and OPC mixes after heating the specimens at elevated temperatures.

The residual compressive strength of OPC and GPC mixes was investigated to be inversely proportional to the temperature and QRD content. The compressive strength of all plain GPC mixes viz. GPC-A0F, GPC-B0F, GPC-C0F, GPC-D0F, and GPC-E0F at 400 °C were decreased by 9%, 13%, 23%, 31%, and 33%, respectively, from the compressive strength at room temperature as shown in Figure 6. The strength of the OPC control mix OPC-0F at 400 °C was decreased by 60%, indicating that GPC mixes are more fire-resistant than the traditional OPC mixes at 400 °C. It can also be seen from Figure 12 that the residual strength of QRD-blended mixes decreased with the increase in QRD content.

The compressive strength of all plain GPC mixes, i.e., GPC-A0F, GPC-B0F, GPC-C0F, GPC-D0F, and GPC-E0F at 800 °C decreased by 57%, 61%, 67%, 71%, and 74%, respectively. The drop in compressive strength increased with the increase in temperature from 400 °C to 800 °C. This strength drop is due to the presence of a large amount of Ca(OH)_2_ and CaCO_3_ in QRD content that is dehydrated and decomposed, respectively, and converted into CaO at temperatures in the range of 600–700 °C [63]. As a result of dehydration and decomposition, H_2_O and CO_2_ are released, causing volume shrinkage and a significant decrease in compressive strength. It can also be one of the reasons that the matrix starts fusing and melting into a near homogeneous phase at 800 °C, which could include the formation of new products [64], resulting in volume reduction. It was observed during fire that spalling of GPC samples having high content of QRD occurred after being exposed to a temperature of about 400 °C.

It was noticed that adding 0.75% SF in OPC- and QRD-blended GPC mixes reduced the loss in compressive strength at high temperatures. The compressive strength of all fiber-reinforced GPC mixes, i.e., GPC-A0.75F, GPC-B0.75F, GPC-C0.75F, GPC-D0.75F, and GPC-E0.75F at 800 °C were decreased by 42%, 43%, 40%, 51%, and 63%, respectively. However, the increase in the volume fraction of SF from 0.75% to 1.5% did not show any improvement in the residual compressive strength at elevated temperatures. This negligible effect on compressive strength could be due to the poor dispersal of SF (1.5% by volume) in highly viscous GPC mixtures.

### 3.6. X-ray Diffraction (XRD)

**Figure 13:**
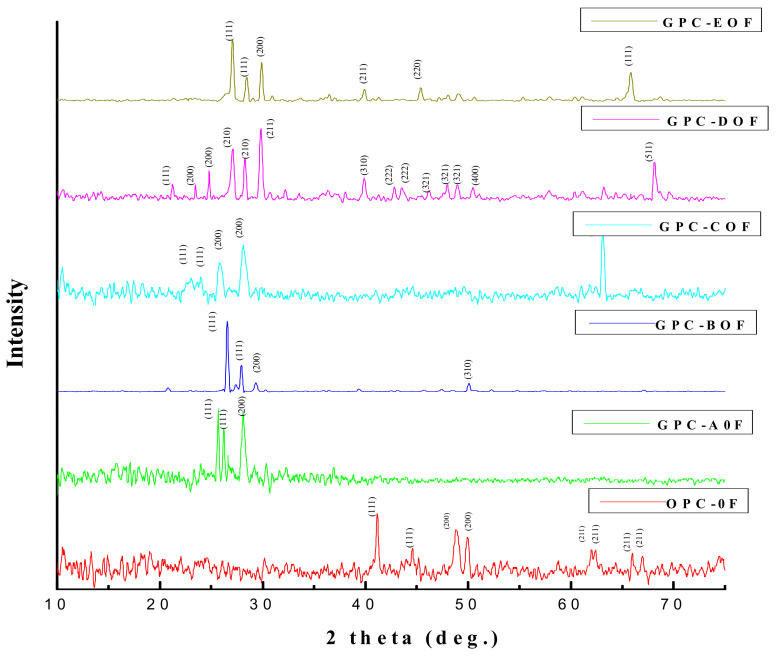
The XRD pattern of OPC and GPC specimens after 28 days.

Figure 13 shows the XRD pattern (2θ = 10–75°) of OPC and GPC mixes (without steel fibers) observed after 28 days of ambient curing. The most significant zone where the reactions occur in the mix is in the range of 2θ = 20–30°. For the specimen GPC-D0F, sharp diffraction peaks are more in this range than all the other samples, including the control mix; which shows that GPC-D0F is highly crystalline. Similarly, in the temperature range 2θ = 40–50°, OPC and GPC-D0F show peaks representing crystalline phases while the other samples, viz. GPC-A0F, GPC-B0F, GPC-C0F, and GPC-E0F are in amorphous phases. Limited periodicity of atoms in the range of 2θ = 60–70° was also present in the samples GPC-C0F, GPC-D0F, and GPC-E0F due to the presence of QRD. The unreacted fly ash and QRD contains crystalline phases such as quartz (SiO_2_), mullite (Al_6_Si_2_O_13_), and maghemite and hematite (Fe_2_O_3_). Some studies on geopolymer materials indicate that a small amount of quartz may have a positive effect on the mechanical properties, and other minerals may have a detrimental effect on the geopolymer [65]. Hence, it can be observed from XRD diffractogram that an increase in the QRD content up to 15% in all GPC mixes resulted in an increase in the compressive strength due to the formation of crystalline phases. This increase is because mechanical properties (compressive, tensile, and flexural) of concrete mixtures increase in the presence of a high amount of calcium-rich species at ambient curing temperature [19,24].

### 3.7. Scanning Electron Microscopy (SEM) and Energy Dispersive Spectroscopy (EDS)

**Figure 14:**
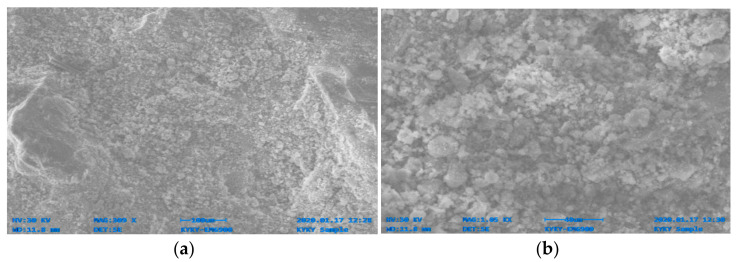
The SEM image of GPC-A0F mix specimen; (**a**) magnification 309×; and (**b**) magnification 1.05×.

**Figure 15:**
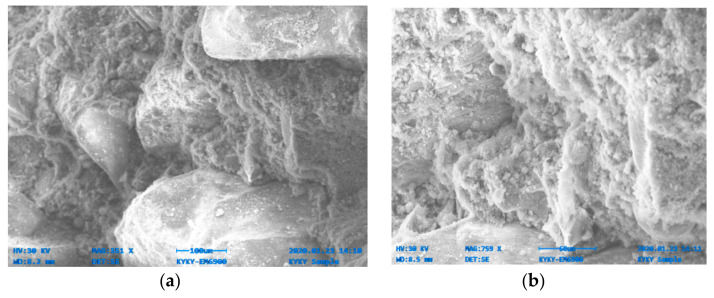
The SEM image of GPC-B0F mix specimen; (**a**) magnification 351×; and (**b**) magnification 759×.

**Figure 16:**
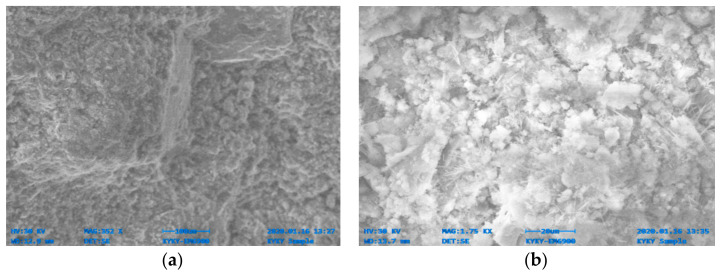
SEM image of GPC-C0F mix specimen; (**a**) magnification 352×; and (**b**) magnification 1.75×.

**Figure 17:**
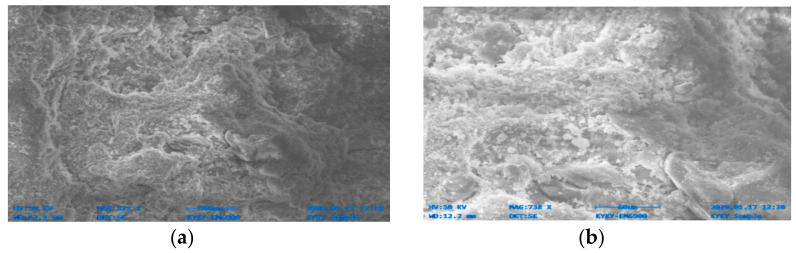
SEM image of GPC-D0F mix specimen; (**a**) magnification 321×; (**b**) magnification 738×.

**Figure 18:**
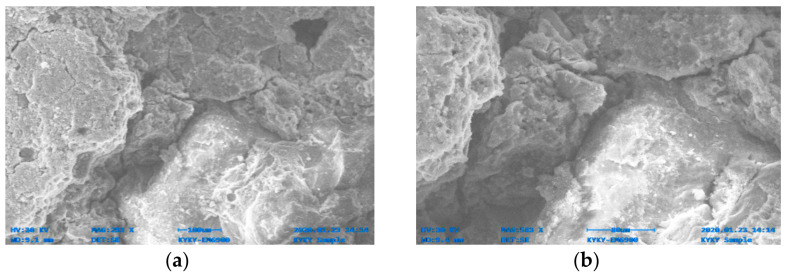
SEM image of GPC-E0F mix specimen; (**a**) magnification 293×; and (**b**) magnification 583×.

**Figure 19:**
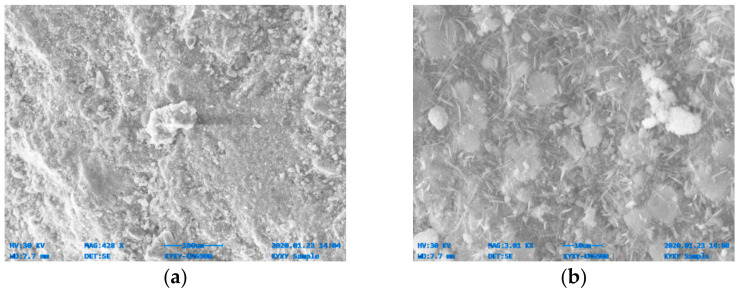
SEM image of OPC-0F mix specimen; (**a**) magnification 428×; and (**b**) magnification 3.01×.

Figure 14, Figure 15, Figure 16, Figure 17, Figure 18 and Figure 19 show the results of SEM micrographs of OPC- and QRD-incorporated GPC specimens.

The microstructure of GPC-D0F prepared with 15% QRD and activated by alkaline solution is denser and less porous than the remaining mixes, i.e., GPC-A0F, GPC-B0F, GPC-C0F, GPC-E0F, and OPC-0F; which shows that GPC-D0F is more compacted. Due to this reason, the strength of GPC-D0F is higher among all mixes. Further, there are no cracks and unreacted particles of FA and SG in the structure of GPC-D0F due to the presence of sufficient CaO in QRD. The SEM image of GPC-E0F shows that by increasing QRD from 15% to 20%, the structure of the hardened mix became porous, causing cracks, which eventually decreased the mechanical strength of the GPC specimens with more than 15% QRD. There are a lot of unreacted particles of FA and SG in the specimens of GPC-A0F, GPC-B0F, and GPC-C0F; which could be the cause of a decrease in the compressive strength. The spherical-shaped FA and angular-shaped SG and QRD particles are in the fuse condition after exposure to elevated temperatures, which decreased the mechanical properties.

**Figure 20:**
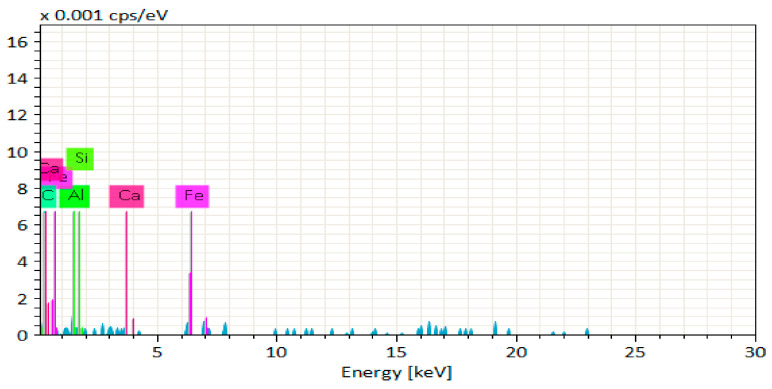
EDS graph of GPC-A0F mix specimen.

**Figure 21:**
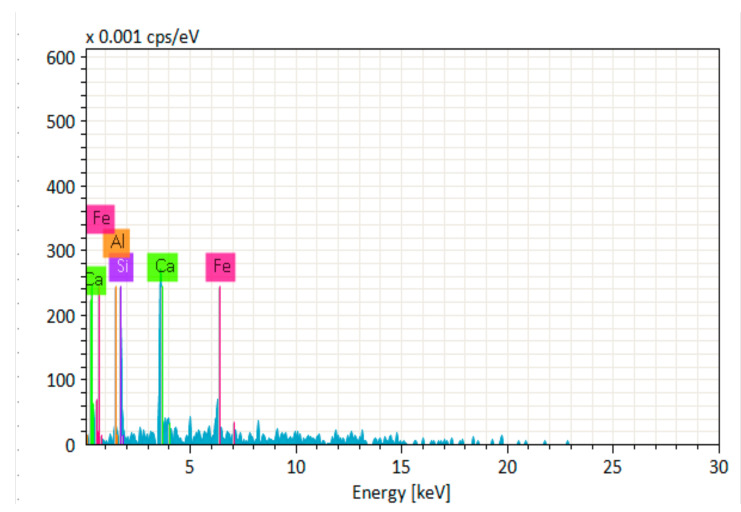
EDS graph of GPC-B0F mix specimen.

**Figure 22:**
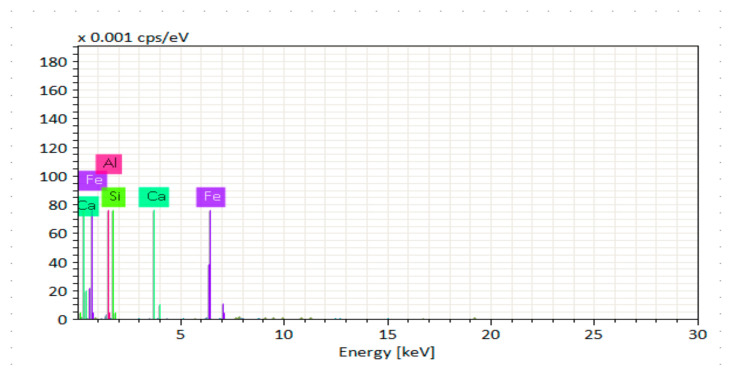
EDS graph of GPC-C0F mix specimen.

**Figure 23:**
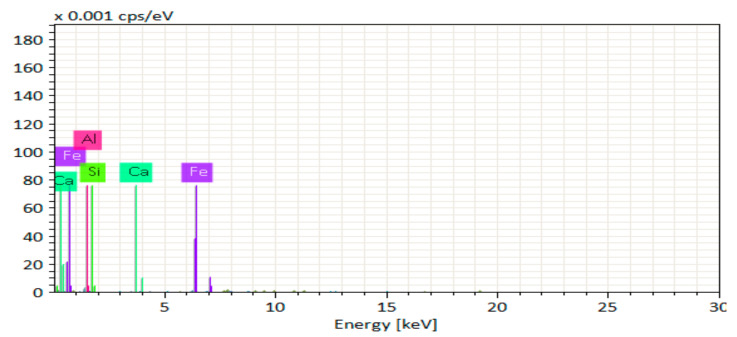
EDS graph of GPC-D0F mix specimen.

**Figure 24:**
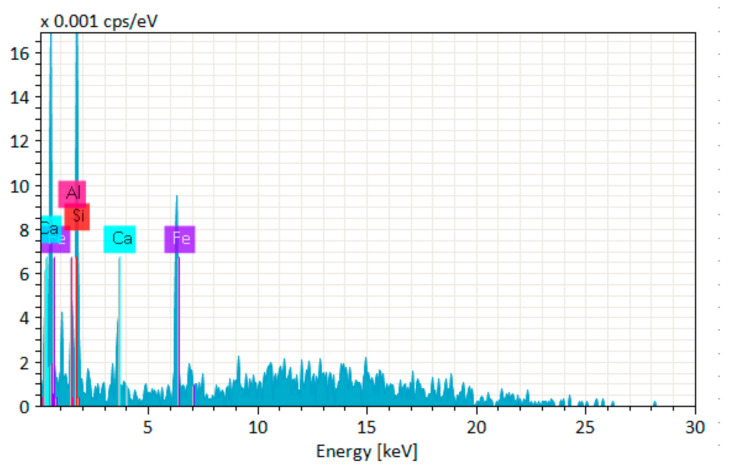
EDS graph of GPC-E0F mix specimen.

**Figure 25:**
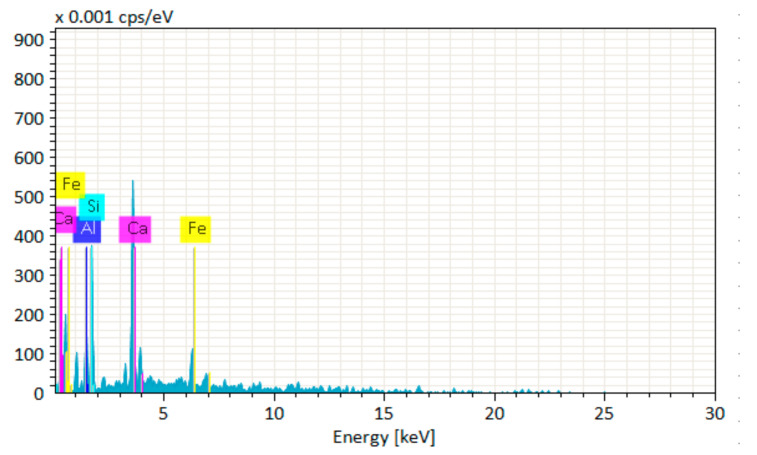
EDS graph of OPC-0F mix specimen.

Figure 20, Figure 21, Figure 22, Figure 23, Figure 24 and Figure 25 show the results of EDS. The presence of elements such as Ca, Si, Al, C, and Fe indicates calcium aluminosilicate hydrate (CASH) in almost all specimens. Therefore, the formation of CASH in GPC-D0F makes the microstructure more compacted and dense, ultimately improving its mechanical properties. The presence of high calcium content in QRD and SG increases the geopolymerization process at ambient temperature which enhances the compressive strength of GPC specimens.

## 4. Conclusions

This paper presented the results of an experimental study conducted to evaluate the influence of QRD and SF inclusion on fresh, mechanical, and residual properties of FA- and SG-based geopolymer concrete at ambient and elevated temperatures. The following key conclusions have been drawn from this study:

1. The workability of GPC mixes decreased by increasing the QRD content and by incorporating SF. QRD has a negative effect on the workability of GPC mixes.
2. The increase in QRD content from 0% to 15% resulted in an increase in the compressive strength of all GPC mixes at 28 and 56 days. The maximum increase in compressive strength was noticed for the 15% replacement level of QRD.
3. The addition of 0.75% SF increased the compressive strength of both OPC and GPC mixes but decreased their workability.
4. The tensile and flexural strength of GPC mixes increased at early ages (maximum up to 28 days) with the increase in QRD content and incorporation of SF. However, a reduction was observed in tensile and flexural strength with the increase of the volume fraction of SF from 0.75% to 1.5%.
5. After heating the GPC specimens at elevated temperatures, the weight loss consistently increased, and residual compressive strength respectively decreased with the increase in QRD content. However, the inclusion of SF reduced the loss in compressive strength of GPC mixes after exposure to elevated temperature.
6. The results of XRD analysis showed that the crystallinity of the geopolymer structure increased by increasing QRD content up to 15% of the total binder content.
7. The SEM analysis exhibited that increasing the QRD content up to 15% improved the mechanical properties due to a dense, less porous, and more compacted microstructure.
8. The EDS analysis showed that high content of calcium compounds improved mechanical properties of GPC specimens

It is worth noting that during the mixing operations, it is difficult to handle GPC at the field due to exothermic reaction and the harmful effect of alkaline solutions on the human body and cloth during use. Therefore, efforts are needed to produce ambient cured GPC using solid activators instead of alkaline solutions, given its wider acceptance in the field.

## Figures and Tables

**Table 1:** The general characteristics of type II OPC used in the study.

Oxides	Results (%)	Physical Characteristics	Results
CaO	64.2	Specific surface	322 m^2^/kg
SiO_2_	22.0	Consistency	30%
Al_2_O_3_	5.50	Initial setting time	1 h 42 min
Fe_2_O_3_	3.50	Final setting time	3 h 55 min
SO_3_	2.90	Specific gravity	3.5
Mg O	2.50	Soundness	No soundness
K_2_O	1.00	Color	Grey
Na_2_O	0.20	-	-
LOI	0.64	-	-

**Table 2:** The chemical composition of QRD, SG, and FA used in the present study.

Oxides	QRD	SG	FA
SiO_2_	9.35%	34.38%	57–65%
Al_2_O_3_	1.64%	12.98%	28–32%
Fe_2_O_3_	1.03%	1.29%	1–4% max
CaO	47.13%	37.33%	1–2%
MgO	1.25%	5.59%	0.50%
K_2_O	0.20%	0.82%	-
Na_2_O	−0.11%	0.29%	1.5 max%
SO_3_	0.08%	0.23% max	4%
SiO_2_:Al_2_O_3_	5.70	2.64	2.03
LOI	38.65%	3.4%	2.9%

**Table 3:** The chemical composition of Na_2_SiO_3_.

Composition	Percentage
Na_2_O	8.93%
SiO_2_	29.8%
Water	61.78%
Density (kg/m^3^)	1400

**Table 4:** The properties of coarse and fine aggregates.

Coarse Aggregate		Fine Aggregate (Sand)	
Moisture content	1.0%	Fineness modulus	2.72
Specific gravity	2.66	Specific gravity	2.74
Water absorption	0.8%	Water absorption	1.25%

**Table 5:** The specifications of hooked end steel fibers.

Entity	Specification
Length	35 mm
Diameter	0.55
Aspect ratio	64
Tensile strength	1345 MPa

**Table 6:** The mix designations of OPC and GPC mixes.

Mix ID	Mix Composition
OPC-F0	100% cement
OPC-F0.75	100% cement + 0.75% steel fibers control mixes
OPC-F1.5	100% cement + 1.5% steel fibers traditional concrete
GPC-AF0	50%FA + 50% SG
GPC-AF0.75	50%FA + 50% SG + 0.75% steel fibers
GPC-AF1.5	50%FA + 50% SG + 1.5% steel fibers
GPC-BF0	50% FA + 45% SG + 5% QRD
GPC-BF0.75	50% FA + 45% SG + 5% QRD + 0.75% steel fibers
GPC-BF1.5	50% FA + 45% SG + 5% QRD + 1.5% steel fibers
GPC-CF0	50% FA + 40% SG + 10% QRD
GPC-CF0.75	50% FA + 40% SG + 10% QRD + 0.75% steel fibers
GPC-CF1.5	50% FA + 40% SG + 10% QRD + 1.5% steel fibers
GPC-DF0	50% FA + 35% SG + 15% QRD
GPC-DF0.75	50% FA + 35% SG + 15% QRD + 0.75% steel fibers
GPC-DF1.5	50% FA + 35% SG + 15% QRD + 1.5% steel fibers
GPC-EF0	50% FA + 30% SG + 20% QRD
GPC-EF0.75	50% FA + 30% SG + 20% QRD + 0.75% steel fibers
GPC-EF1.5	50% FA + 45% SG + 5% QRD + 1.5% steel fibers

**Table 7:** The detail of mix proportions of OPC and GPC mixtures.

Group ID	Mix No.	Mix ID	B	C	Concrete Mixture Quantity (kg/m^3^)														
					Binders			SF	AL/B Ratio	W/C Ratio	Molarity of SH	SS/ SH Ratio	SH	SS	S	CA	CA	SPs	Water
					FA	SG	QRD									20 mm	10 mm		
OPC	1	OPC-0F	400	400	-	-	-	-	-	0.35	-	-	-	-	680	751	340	10	140
	2	OPC-0.75F	400	400	-	-	-	58.5	-	0.35	-	-	-	-	680	752	340	10	140
	3	OPC-1.5F	400	400	-	-	-	117	-	0.35	-	-	-	-	680	753	340	10	140
GPC-A	4	GPC-A0F	400	-	200	200	0	-	0.5	-	12	1.5	80	120	680	751	340	11	35
	5	GPC-A0.75F	400	-	200	200	0	58.5	0.5	-	12	1.5	80	120	680	752	340	18	35
	6	GPC-A1.5F	400	-	200	200	0	117	0.5	-	12	1.5	80	120	680	753	340	20	35
GPC-B	7	GPC-B0F	400	-	200	180	20	-	0.5	-	12	1.5	80	120	680	754	340	12	35
	8	GPC-B0.75F	400	-	200	180	20	58.5	0.5	-	12	1.5	80	120	680	755	340	17	35
	9	GPC-B1.5F	400	-	200	180	20	117	0.5	-	12	1.5	80	120	680	756	340	21	35
GPC-C	10	GPC-C0F	400	-	200	160	40	-	0.5	-	12	1.5	80	120	680	757	340	14	35
	11	GPC-C0.75F	400	-	200	160	40	58.5	0.5	-	12	1.5	80	120	680	758	340	20	35
	12	GPC-C1.5F	400	-	200	160	40	117	0.5	-	12	1.5	80	120	680	759	340	22	35
GPC-D	13	GPC-D0F	400	-	200	140	60	-	0.5	-	12	1.5	80	120	680	760	340	14.5	35
	14	GPC-D0.75F	400	-	200	140	60	58.5	0.5	-	12	1.5	80	120	680	761	340	21	35
	15	GPC-D1.5F	400	-	200	140	60	117	0.5	-	12	1.5	80	120	680	762	340	23	35
GPC-E	16	GPC-E0F	400	-	200	120	80	-	0.5	-	12	1.5	80	120	680	763	340	14.5	35
	17	GPC-E0.75F	400	-	200	120	80	58.5	0.5	-	12	1.5	80	120	680	764	340	22	35
	18	GPC-E1.5F	400	-	200	120	80	117	0.5	-	12	1.5	80	120	680	765	340	24	35

**Note:** W (water): B (binder); C (cement): OPC (ordinary portland cement); SF (steel fibers); AL (alkaline solution); QRD (quarry rock dust); SG (ground granulated blast furnace); FA (fly ash); SH (sodium hydroxide); SS (sodium silicate); SP (superplasticizers); S (sand); CA (coarse aggregates).

## Data Availability

Not applicable.

## Abbreviations

QRD	quarry rock dust
SG	ground granulated blast furnace slag
FA	fly ash
SP	superplasticizer
SF	steel fiber
W/C	water-cement ratio
